# Supplementary material for: The genome sequence of the Dotted Oak Knot-horn moth, Phycita roborella (Denis & Schiffermüller) 1775
Source: Wellcome Open Res. 2024 Nov 6;9:653. [Version 1] doi: 10.12688/wellcomeopenres.23206.1 (PMC11803376; doi:10.12688/wellcomeopenres.23206.1)
Supplement: Peer review history

# Reviewer report: Reviewer response for version 1

**DOI:** 10.21956/wellcomeopenres.25558.r111795

**Published:** 2024-12-13

**Authors:** Savarimuthu Ignacimuthu, Sivasankaran Kuppusamy

**Affiliations:** 1 St Xavier's College, Palayamkottai, Tamil Nadu, India; 2 Entomology Research Institute, Loyola College, Chennai, Tamil Nadu, India

Authors have sequenced chromosome-level genome of Dotted Oak Knot-horn moth, *Phycita roborella* ([Denis & Schiffermüller], 1775).  Through the comprehensive data, a total of 33 chromosome conformation have been done by the authors based on the assembly. Illumina and PacBio have been used for long read sequencing, appropriate software was used for assembling and annotation of the sequence.

**Minor comments on the manuscript**

 In the title of the article the square bracket hasn't been provided for the species' authors. The square bracket can be included in the title i.e., ([Denis &Schiffermüller], 1775)

 The authors have given the complete form of the genus name *Phycita roborella* throughout the article. The genus name may be given in full the first time, and then in abbreviated form later on, such as *P. roborella*

 Through genome assembly authors would have received the protein-coding genes and gene transcripts. The protein-coding genes and gene transcripts haven't been included in the text and table. Explain.

 Above all, I confirm that the manuscript meets the necessary scientific standard and is suitable for indexing.

Are sufficient details of methods and materials provided to allow replication by others?

Yes

Is the rationale for creating the dataset(s) clearly described?

Yes

Are the datasets clearly presented in a useable and accessible format?

Yes

Are the protocols appropriate and is the work technically sound?

Yes

Reviewer Expertise:

Molecular biology

We confirm that we have read this submission and believe that we have an appropriate level of expertise to confirm that it is of an acceptable scientific standard.

# Reviewer report: Reviewer response for version 1

**DOI:** 10.21956/wellcomeopenres.25558.r111798

**Published:** 2024-11-16

**Authors:** Michael Hiller

**Affiliations:** 1 Senckenberg Nature Research Society, Frankfurt Am Main, Germany

A high-quality genome assembly of the moth *Phycita roborella* is reported. HiFi and HiC data was used to assemble a very complete chromosome-level genome with a very high base accuracy. Since other moth species have a similar appearance, the identity of the sequenced individual was validated by its COI DNA barcode.

**I found one likely error and have a few suggestions:**

- The text says 4.07 Gb HiFi data was generated but Table 1 lists 27.42. I assume the table 1 correct, given the assembly size of 710 Mb.
- I don't understand the last part of this sentence "... and the adults fly at dusk from June to September in one or perhaps occasionally two generations, coming to light, flowers and sugar." What is meant with sugar? Please clarify.
- Maybe introduce the common name (snout moths or grass moths) of Pyralidae in the introduction.
- If possible and images are available, the differences between *Phycita *and *Acrobasis*/ *Delplanqueia* that are described in the text could be illustrated in a panel of Figure 1, but I understand that this is a genome note of course.

Are sufficient details of methods and materials provided to allow replication by others?

Yes

Is the rationale for creating the dataset(s) clearly described?

Yes

Are the datasets clearly presented in a useable and accessible format?

Yes

Are the protocols appropriate and is the work technically sound?

Yes

Reviewer Expertise:

genome assembly, comparative genomics

I confirm that I have read this submission and believe that I have an appropriate level of expertise to confirm that it is of an acceptable scientific standard.
